# Cross-specialty Collaboration and Education for Neurosurgical Trainees: Teaching Arterial Line Placement Under Ultrasound Guidance

**DOI:** 10.7759/cureus.5900

**Published:** 2019-10-13

**Authors:** Zachary Risler, Mark A Magee, Elinna Hatter, Kelly Goodsell, Ashwini Sharan, Arthur K Au, Resa E Lewiss

**Affiliations:** 1 Emergency Medicine, Thomas Jefferson University Hospital, Philadelphia, USA; 2 Neurosurgery, Thomas Jefferson University Hospital, Philadelphia, USA

**Keywords:** medical eduction, procedural training, interprofessional collaboration, arterial line, ultrasound (us)

## Abstract

Introduction

Interprofessional collaboration (IPC) increases patient safety. IPC is learned through task-based exercises, such as ultrasound (U/S)-guided arterial lines. We set out to teach U/S-guided arterial lines as a framework to improve IPC between emergency medicine and neurosurgery residents. The objectives of the study were to provide a U/S session to teach the proper arterial line placement technique, to assess post-workshop arterial line placement competency and attitude toward U/S for procedural guidance, and to improve interdepartmental relationships through IPC.

Methods

The course was completed in 2018 and consisted of pre-workshop assignments, the workshop, a competency assessment, and a post-workshop survey for neurosurgical residents. After a didactic and hands-on training session, trainees completed a simulated U/S-guided arterial line placement. Trainees then completed a post-workshop assessment.

Results

There were a total of 21 participants out of 24 total residents, an 87.5% participation rate. Prior to the workshop, on a 5-point Likert scale, where 1 is not at all likely and 5 is very likely, the residents reported they would use U/S 1.7/5, with 57% of respondents answering 1 out of 5. After the workshop, on the same Likert scale, the residents reported using U/S first 3.6/5 (P < 0.05) with 52% of the respondents answering 4 out of 5. After the course, the belief that the landmark technique is non-inferior decreased to 28.6% of respondents.

Conclusions

The overall goal of this workshop was to improve patient care through continuing education. Using IPC as the framework, the workshop significantly increased the reported likelihood of using U/S for arterial line placement.

## Introduction

Interprofessional collaboration (IPC) education occurs when members of two or more health or social care professions work together to improve the care of patients. Such collaboration increases patient safety and improves patient management [1-4]. The de-siloing of care brings departments, patient groups, and medical providers together to share in the process of improving care. IPC is a core competency for neurological surgery residency who, as mandated by the Accreditation Council for Graduate Medical Education (ACGME), are expected to “work in interprofessional teams to enhance patient safety and improve patient care quality” [5]. IPC is learned through task-based exercises, simulation, and classroom learning. Ultrasound (U/S)-assisted vascular access is standard teaching and practice through an emergency medicine residency. Not only does U/S guidance for peripheral venous line placement increase the rate of successful cannulation in difficult access patients, but also the same is true for central venous access line placement [6-9]. Relatedly, U/S guidance for arterial line placement is well-supported by the literature. However, U/S guidance is often overlooked and under-implemented [10]. As compared to the palpation technique, U/S-guided arterial line placements require less time, fewer attempts, and are associated with fewer complications [10-11]. One meta-analysis showed a 71% improvement in the likelihood of first-pass success of arterial line placement with U/S guidance [12]. Practitioners are less familiar with the U/S technique than standard palpation for arterial lines [12]. At our institution, neurosurgery trainees place arterial lines; however, this is not routinely done with U/S guidance. 

Introduction to the need for an IPC workshop

A neurosurgical trainee, without familiarity with the U/S machine use, was placing an U/S-guided arterial line in the emergency department. The procedure, while ultimately successful, could have been improved. We saw this as an ideal IPC opportunity with the goal of providing education for patient safety and patient-centered care. We organized an interprofessional workshop to educate neurosurgery trainees on the U/S-guided arterial line placement technique. As a secondary outcome, we attempted to improve departmental relationships and encourage a train-the-trainer sustainable program. Given the current ACGME and graduate medical education (GME) requirements, we saw U/S as an exceptional medical technology to model IPC for patient care.

## Materials and methods

The two-hour workshop was designed specifically for neurosurgical residents and was completed at the beginning of the 2018 academic year. The workshop consisted of pre-workshop assignments, a workshop, a competency assessment, and a post-workshop follow-up. Five emergency medicine U/S instructors with U/S fellowship experience taught 21 out of a possible 24 neurosurgical residents. Trainees were asked to complete flipped classroom assignments compiled from Taming the SRU (Shock Resuscitation Unit) and the Ultrasound Podcast websites [13-14]. The assignment included online lectures and readings. The workshop started with a 10-minute didactic lecture discussing U/S-guided technique and procedure setup. The lecture was followed by a hands-on session. At each station, the residents were encouraged to familiarize themselves with the U/S machine knobs and buttons. The residents scanned normal anatomy to identify the radial artery and surrounding structures on their colleagues. They then practiced cannulation of a vessel and catheter placement using static simulation task trainers.

Competency assessment

Once trainees felt comfortable, they completed a simulated U/S-guided arterial line placement under the supervision of a U/S faculty educator. A standard checklist methodology was used. The emphasis for this competency was U/S guidance for an arterial line as the placement of an arterial line is within the scope of practice for neurosurgeons in training. The trainees placed the instructor’s arm into a proper position. They were required to scan to identify proper anatomy and placement location. They then moved to a simulated arterial line model and under U/S guidance placed an arterial line catheter. The residents verbalized actions that were not immediately simulated, such as lidocaine injection and suturing the arterial line in place. The instructor then went through the checklist with the resident to ensure the entire procedure was completed correctly. The Jefferson Office of Human Research approved this study. All adult participants provided written informed consent to participate.

## Results

**Figure 1:**
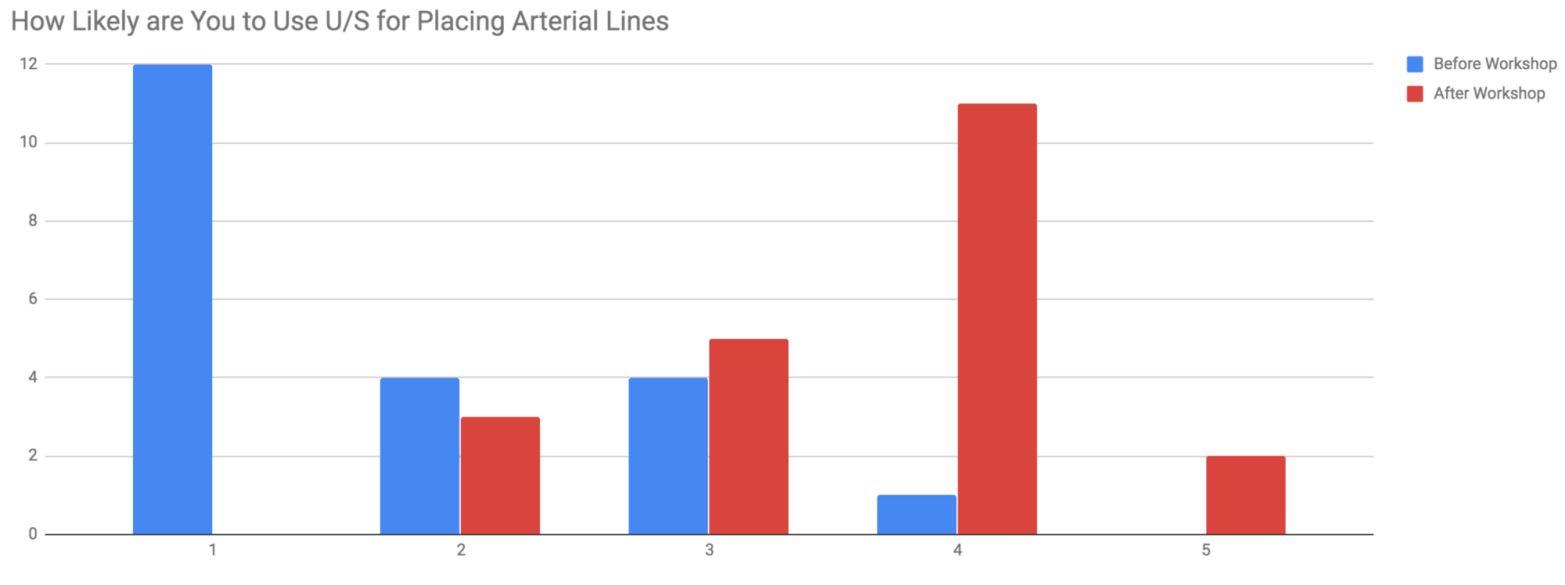
Likeliness of ultrasound (U/S) use for arterial line placement before and after the workshop on a 5-point Likert scale

**Figure 2:**
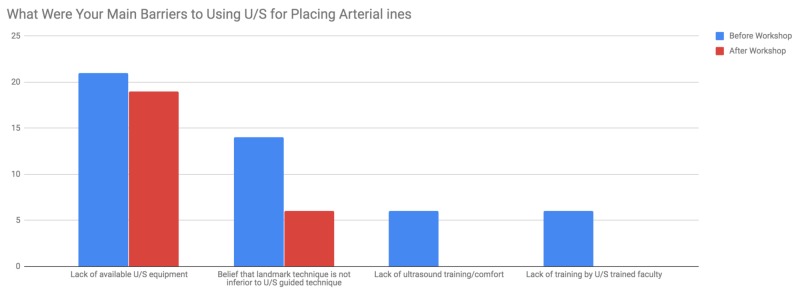
Main barriers to ultrasound (U/S) use for arterial line placement before and after the workshop

A pre-workshop survey of the neurosurgery residents found that none had used U/S first for arterial line placement. There were a total of 21 trainees out of a possible 24 for an 87.5% participation rate in both the workshop and the survey. Prior to the workshop, we found that on a 5-point Likert scale, where 1 is not at all likely and 5 is very likely, the residents reported they would use U/S 1.7 out of 5. Of the respondents, 57% answered 1 out of 5. The residents shared their perceived barriers to using U/S prior to the course and lack of access to U/S equipment was the largest barrier to U/S guidance (100% of respondents), followed by the belief that the landmark technique is non-inferior (66.7% of respondents). After the workshop, on the same Likert scale, the residents reported using U/S first 3.6 out of 5 (P < 0.05) with 52% of respondents answering 4 out of 5 (Figure 1). After the course, the belief that the landmark technique is non-inferior decreased to 28.6% of respondents. Furthermore, the lack of training and lack of comfort decreased as barriers to using U/S (Figure 2).

## Discussion

The U/S workshop increased the reported likelihood that neurosurgical residents would place arterial lines utilizing U/S. While the literature supports that arterial line placement under U/S guidance takes less time and fewer needle sticks, it is still not the standard of care. In contrast, the use of ultrasound guidance in central venous access is almost always done under U/S guidance which lends credence to the fact that U/S guidance is safer for procedures. 

A meta-analysis by White et al. found a high level of evidence supporting the use of ultrasound guidance in radial artery cannulation [15]. They found that ultrasound in adults significantly increased first attempt success and, therefore, significantly reduced the number of attempts. Another paper by Hansen et al. found that U/S guidance using the real-time needle guidance for radial artery catheterization significantly improved successful clinically relevant aspects of the procedure, such as first-pass success and fewer overall attempts [16]. Our workshop taught neurosurgical residents ultrasound guidance and will hopefully lead to increasing ultrasound use and, in turn, better patient care through decreased attempts and higher first-pass success. 

We also hope that the use of small workshops between departments will increase collaboration. Based on prior studies, the top five reasons for engaging in interprofessional collaboration were to improve collaboration, communication, patient safety, healthcare quality, and attitudes towards teamwork [17]. We hope that small steps towards collaboration, such as workshops, will open the silos of our current medical practice.

There are some limitations to this study. We describe a one-time training conducted on a limited number of trainees. There has not been a follow-up assessment or competency performed to assess the true integration of U/S into practice. Additionally, some of the neurosurgery residents attended a portion rather than the whole session. The respondents also answered the questions within a few weeks of the course, so it is unclear if they will truly retain the information presented.

## Conclusions

The overall goal of this workshop was to improve patient care through continuing education. This objective was accomplished using interprofessional collaboration between the neurosurgical residency and the point-of-care ultrasound division of the Department of Emergency Medicine. The Institute of Medicine regards IPC as a way to increase collaboration between disciplines. While the focus of this workshop was narrow, the overall goal of our division was to increase collaboration through ultrasound education to enhance patient care at our institution. We have only just begun this process, as more research is necessary to test our broader goals.
